# Strategies of Pathogens to Escape from NO-Based Host Defense

**DOI:** 10.3390/antiox11112176

**Published:** 2022-11-03

**Authors:** Giovanna De Simone, Alessandra di Masi, Paolo Ascenzi

**Affiliations:** 1 Dipartimento di Scienze, Università Roma Tre, 00146 Roma, Italy; 2 Laboratorio Interdipartimentale di Microscopia Elettronica, Via della Vasca Navale 79, 00146 Roma, Italy

**Keywords:** nitric oxide, heme-proteins, *S*-nitrosylation, pathogens

## Abstract

Nitric oxide (NO) is an essential signaling molecule present in most living organisms including bacteria, fungi, plants, and animals. NO participates in a wide range of biological processes including vasomotor tone, neurotransmission, and immune response. However, NO is highly reactive and can give rise to reactive nitrogen and oxygen species that, in turn, can modify a broad range of biomolecules. Much evidence supports the critical role of NO in the virulence and replication of viruses, bacteria, protozoan, metazoan, and fungi, thus representing a general mechanism of host defense. However, pathogens have developed different mechanisms to elude the host NO and to protect themselves against oxidative and nitrosative stress. Here, the strategies evolved by viruses, bacteria, protozoan, metazoan, and fungi to escape from the NO-based host defense are overviewed.

## 1. Introduction

Nitric oxide (NO) is a pivotal messenger molecule able to control, via the induction of cyclic guanosine monophosphate (cGMP) production, a wide range of biological processes including vasomotor tone, neurotransmission, and immune response [1,2,3,4,5,6,7,8,9,10,11]. Furthermore, NO controls gene transcription and mRNA translation by modulating the transcriptional activity of the iron-responsive elements [12,13,14,15].

**Figure 1:**
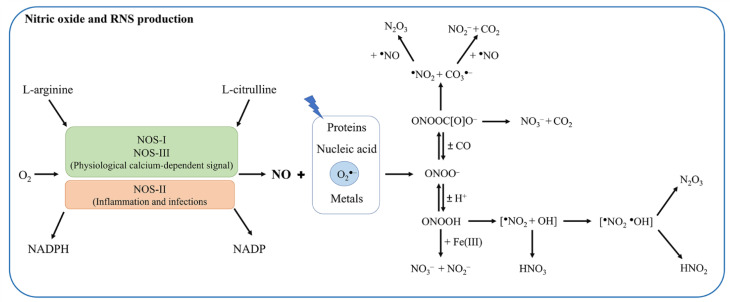
Overview on NO and reactive nitrogen species (RNS) production. NO can be produced from the oxidation of L-arginine in the presence of O_2_, NADPH, and various co-factors. This reaction is catalyzed by the constitutive NOS-I (nNOS) and NOS-III (eNOS) enzymes, as well as by the inducible NOS-II (iNOS). Once produced, NO can interact with O_2_, metals, nucleic acids, and proteins as well as with O_2_^●−^, generating peroxynitrite (ONOO^−^). ONOO^−^ reacts with CO_2_ to form 1-carboxylato-2-nitrosodioxidane (ONOOC[O]O^−^), which decays by homolysis of the O–O bond to yield the reactive species nitrogen dioxide (^●^NO_2_) and trioxocarbonate (CO_3_^●−^).

**Figure 2:**
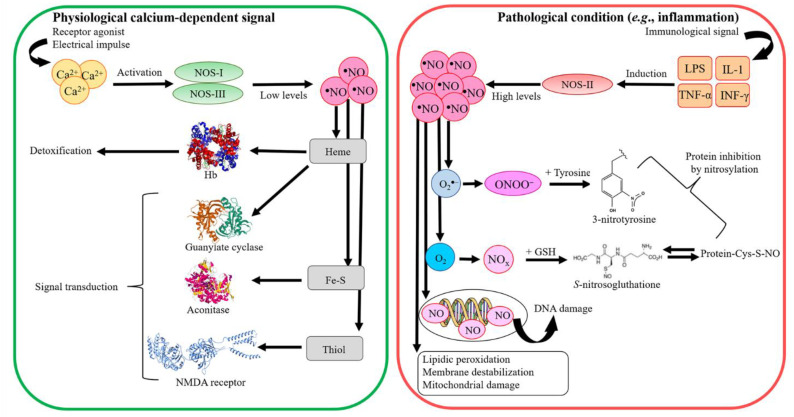
Effects of NO under physiological and pathological conditions. The activity of the constitutive NOS-I and NOS-III enzymes depends on the intracellular calcium levels. On the contrary, NOS-II expression is calcium-independent and is induced under inflammatory conditions. At low physiological concentrations (green box), NO produced by constitutive NOS-I and NOS-III acts as a physiological modulator of protein activities (e.g., reacting with heme groups, iron–sulfur cluster, and thiol groups). At high pathological NO concentration (red box) (e.g., during inflammation and infections) and in the presence of oxidative agents (i.e., O_2_^●−^), NO can trigger irreversible damages to biological macromolecules (i.e., DNA oxidation, protein nitrosylation, oxidation of thiol groups, and lipid peroxidation) and can induce cell overall damage (e.g., inhibition of cytochrome *c* oxidase (CcO) activity, inactivation of mitochondrial respiration, cell membrane alteration, and induction of cell death and differentiation). The three-dimensional structures of Hb (PDB ID: 1NQP) [52], guanylate cyclase (PDB ID: 4NI2) [53], aconitase (PDB ID: 2B3Y) [54], and NMDA receptor (PDB ID: 6IRA) [55] were drawn using UCSF-Chimera [56].

NO is generated in a variety of cell types by the concomitant conversion of L-arginine to L-citrulline through a reaction catalyzed by at least three distinct isoforms of NO synthase (NOS) [16,17,18,19,20,21,22,23,24]. In mammals, NOS-I and NOS-III are constitutively expressed calcium-dependent enzymes also known as neuronal NOS (nNOS) and endothelial NOS (eNOS), respectively, since they have been originally isolated in neuronal and vascular endothelial cells. NOS-II is a calcium-independent inducible form (iNOS) whose expression is induced by pathological conditions (e.g., inflammation and infection). NO produced by NOS-II contributes to the antimicrobial activity of macrophages and represents the fraction of exhaled NO, which is considered an inflammatory marker [22,23,25,26]. As NOS-II acts as a cytotoxic effector and immune modulator [22,23,25,26], inducible NO production needs a tight control as it can be detrimental to the host [25,27,28,29,30,31,32] (Figure 1). Indeed, free radical NO (^●^NO): (i) forms the nitrosonium cations (NO^+^); (ii) generates the nitroxyl anion (NO^−^); (iii) auto-oxidizes into dinitrogen trioxide (N_2_O_3_), which rapidly is converted to nitrite (NO_2_^−^); and (iv) reacts with the superoxide radical anion (O_2_^●−^) and H_2_O_2_-producing reactive nitrogen species (RNS) [30]. Indeed, the reaction of ^●^NO with O_2_^●−^ leads to the powerful oxidant peroxynitrite (ONOO^−^/HOONO), which is more reactive than its precursors ^●^NO and O_2_^●−^ [33,34,35,36]. Peroxynitrite reacts with carbon dioxide (CO_2_) to form 1-carboxylato-2-nitrosodioxidane (ONOOC[O]O^−^), which decays by homolysis of the O–O bond to yield the reactive species nitrogen dioxide (^●^NO_2_) and trioxocarbonate (CO_3_^●−^); of note, CO_3_^●−^ is a stronger oxidant than ^●^NO_2_ and ONOO^−^ [34,35,37] (Figure 1). Most reactions of CO_3_^●−^ are one-electron oxidations with preference for Tyr and Trp residues. In addition, NO auto-oxidation leads to the formation of N_2_O_3_ that induces the nitrosation of Cys residues (Figure 2). In turn, *S*-nitrosothiols (RSNOs) are involved in cell signaling and regulatory processes (e.g., bronchodilation and neuroprotection). Furthermore, NO binds to transition metals (e.g., the heme-Fe atom). In the vasculature, NO is rapidly converted to NO_3_^−^ by oxyhemoglobin (Hb(II)O_2_), which contributes to the very short half-life of NO (0.1–2.0 s) and in turn acts as a local modulator of vasodilation. Moreover, NO participates to O_2_ transport by delivery from hemoglobin (Hb) [22,30,38,39,40,41,42,43,44] (Figure 2).

NO, nitrite, nitrate, and peroxynitrite are bactericidal molecules that play a central role in the ability of activated macrophages to kill pathogens [45]. Macrophages respond to cytokines and recognize several molecules exhibiting pathogen-associated molecular patterns (PAMPs) through specific receptors (e.g., primarily Toll-like receptors (TLRs) and Nod-like receptors (NLRs)) [45].

Much evidence supports the critical role of NO in the virulence and replication of many viruses, bacteria, and parasites, thus representing a general mechanism of host defense [26,31,46,47,48]. However, pathogens have developed several mechanisms to elude the host barriers to protect themselves against oxidative and nitrosative stress [32,49]. Under anaerobic conditions, many environmental commensal (e.g., *Escherichia coli*) and pathogenic bacteria (e.g., *Mycobacterium tuberculosis*, *Salmonella typhimurium*) reduce the NOS-II-derived NO and dietary nitrate or nitrite to NO via nitrate respiration, nitrate dissimilation, or denitrification [45]. On the contrary, *Listeria monocytogenes* uses the host-derived NO as a gateway to enter the host [45,50,51]. Here, the strategies evolved by viruses, bacteria, protozoan, metazoan, and fungi to escape from the NO-based host defense are reviewed.

## 2. Differences in Macrophage NO Production and Regulation in Mice and Humans

In macrophages, an important component of the host immune defense is represented by NOS-II-mediated NO production [57]. Murine macrophages produce large amounts of NO via NOS-II, consuming most of the L-arginine generated by arginase, an enzyme inactive in human macrophages [58,59,60,61,62]. Moreover, in murine macrophages the transcription of NOS-II is induced by IFN-γ, IFN-α/β, and microbial products (e.g., lipopolysaccharide (LPS)). Furthermore, murine macrophages synthetize the tetrahydrobiopterin (BH4) cofactor that is necessary to stabilize and promote NOS-II activity [45,59,61,63]. In contrast, human, rabbit, Syrian hamster, and goat macrophages do not synthesize BH4 [61,64,65].

In murine macrophages, LPS and INFs induce NOS-II transcription by the activation of the nuclear factor NF-kB and the ISGF3-complex (composed of STAT1, STAT2, and IRF9) [45,66,67]. In human cells, the expression of NOS-II depends on: (i) the cell type, (ii) the activation NF-kB and STAT1 by LPS, INFs, and cytokines, (iii) structural changes in the promoter of NOS-II, and (iv) post-translational histone modifications [62,68]. For example, human alveolar macrophages stimulated with IFN-γ and LPS are not able to express NOS-II, as the NOS-II promoter is subjected to epigenetic gene silencing via CpG methylation, histone modifications, and chromatin compaction [45,57,69,70,71].

Differences in the regulation of the NO pathway have been reported in rats and humans [57]. Arteries from rats subjected to bacterial peritonitis or infused with LPS show a contractile hypo-responsiveness independent from NO production; in contrast, in vitro activation of the aortic smooth muscle cells with a variety of molecules (e.g., LPS, IL-1β, and TNF-α) induces NO production [57]. Similarly, human vessels treated with cytokines do not display any change in NOS mRNA levels, while isolated human aortic smooth muscle cells stimulated with LPS plus cytokines increase NOS-II mRNA expression [57].

Interestingly, both in murine and human cells the mRNA and protein expression levels of NOS-II are affected by microRNAs (miRNAs) [45]. Most of the identified miRNAs (including miR-125a-5p, miR-146a, miR-149, and miR-155) indirectly regulate the expression of NOS-II by blocking the expression of transcription factors (e.g., IRAK-1, NF-kB repressing factor, and SOCS-1) [45,72]. In contrast, miR-939, miR-26a, and miR-146a expressed in human hepatocytes, T cells, lymphoma cells, and mouse renal carcinoma cells, respectively, cause a downregulation of NOS-II expression and NO production by interacting with the 3′-UTR of NOS-II mRNA [72,73,74].

## 3. Antiviral Action of NO

The antiviral action of NO has been reported for several DNA and RNA virus families including *Picornaviridae*, *Flaviviridae*, *Coronaviridae*, *Rhabdoviridae*, *Reoviridae*, *Retroviridae*, *Parvoviridae*, *Herpesviridae*, and *Poxviridae* [30,31,75,76,77,78,79]. In fact, NO affects: (i) the transcription process, (ii) the post-transcriptional enzyme activity, and (iii) the protein assembly [75,78,80,81,82,83,84]. NO can prevent virus replication by the *S*-nitrosylation of Cys residues present in viral proteases, reductases, and reverse transcriptases [30,31,75,79] and/or by modulating early transcript levels through the introduction of strand breakages and deamination of adenine and guanine bases [85].

**Figure 3:**
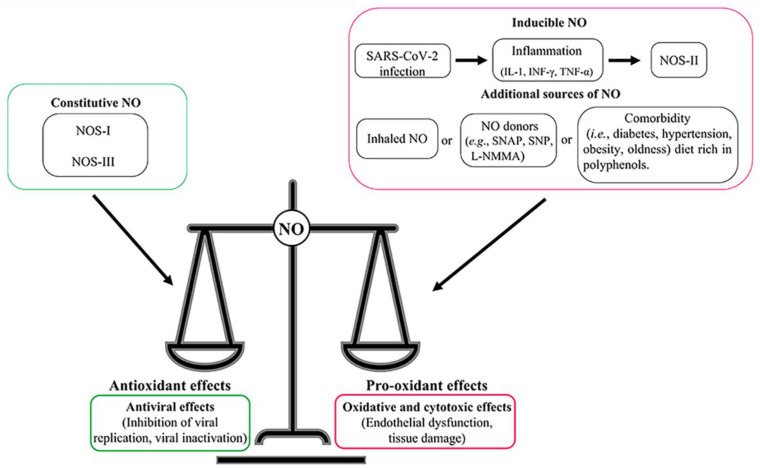
Role of NO during the viral infection. Constitutive NO exerts an antiviral activity controlling infection whereas the inducible and exogenous NO contributes to inflammation and pro-oxidants effects (e.g., tissue damage and cell death).

During viral infection, the effect of NO may depend on both the time of infection and the pathogenic mechanism. Once viruses infect the host cells, the increased NO production can upregulate NOS-II through three pathways [30] (Figure 3). The first pathway is dependent on TLRs, which are expressed on the membrane of macrophages, T and B lymphocytes, and non-immune epithelial cells. TLRs recognize the viral DNA or RNA and activate both NF-κB and AP1 [78]. The second pathway involves IFN-γ, which is produced by lymphocytes and activates STAT-1 [30]. The third pathway involves viral double-stranded RNAs that induce IFNs and R-protein kinases (PKR) expression [86] (Figure 2 and Figure 3).

In non-respiratory infections (e.g., hepatitis C, dengue fever, and herpes simplex), NO production is typically associated with pathogenic effects [79,87,88,89,90]. In fact, a positive correlation between NOS-II activity and liver damage has been found in patients chronically infected by hepatitis C virus [88,89]. Moreover, the serum of humans affected by dengue fever shows increased NO levels that, by inducing dilatation of the blood vessels, could be relevant in the evolution of the infection into a severe hemorrhagic form [90]. NO also displays a role in the pathogenesis of herpes simplex virus type 1 (HSV-1) as demonstrated by the fact that mice infected with HSV-1 and treated with the NOS-II inhibitor L-NMMA show a higher survival rate and a better lung compliance compared to the untreated control group [79,87,91].

In the respiratory infections (e.g., common cold caused by human rhinovirus (HRV) and influenza A virus (IAV), MERS and COVID-19 severe acute respiratory syndrome coronavirus (SARS-CoV-2) infections), the NO production may provide a first line of defense against viruses, acting as an innate immune response component [79]. Patients infected with HRV display NOS-II upregulation that results in high levels of exhaled NO (3- to 5-fold higher compared to the baseline), which in turn mitigates HRV-induced symptoms and contributes to the disease resolution [79,92,93]. Similarly, high NO levels have been also observed in patients affected by IAV infection; however, NO does not influence IAV patient symptoms [94] (Figure 3).

Viruses might avoid the NO-based antiviral effect though different strategies: (i) by inhibiting NOS-II activity during the early stages of the viral infection [95], (ii) by lacking the nitrosylation consensus sequence in the viral protease [96,97], and (iii) by infecting hosts with pre-existing endothelial dysfunction(s) and reduced NO bioavailability [32,98,99,100]. Viruses that can interfere with NO synthesis seem to replicate more rapidly [101]. In this regard, the adenovirus E1A reduces NOS-II transcription thus eluding the host immune responses. Similarly, in vitro studies suggest that HSV-1 inhibits the activity of constitutive NOS in the brain during the early stages of infection and in the presence of circulating glucocorticoids. Brain NOS inhibition by HSV-1 may play a role in the neuronal viral invasion and in the activation of the adrenocortical system [95]. In vitro evidence shows that *Coxsackievirus* has a selective advantage when the 3C^pro^ viral protease lacks the consensus nitrosylation motif (i.e., Xxx(Lys,Arg,His)Cys(Asp,Glu)) [96,97].While NO does not inhibit genome replication of viruses encoding serine proteases (e.g., *Alphavirus*), it blocks the replication of viruses encoding cysteine proteases (e.g., *Picornavirus* and *Coronavirus*) [84,97,102]. Indeed, in silico and in vitro studies suggest that *S*-nitrosylation of cysteine proteases (e.g., TMPRSS2 and 3CL) can play a key role in the inhibition of the SARS-CoV-2 viral life cycle [103,104,105,106,107,108]. Indeed, *S*-nitrosylation of Cys281 and/or Cys348 residue(s) of TMPRSS2 impairs SARS-CoV-2 entry into lung epithelial cells [108]. In addition, *S*-nitrosylation of the Cys145 residue of the 3CL protease interrupts the maturation of the viral polyproteins that are necessary for replication [105,108]. Of note, in vitro studies suggest that the NO donor sodium nitroprusside can nitrosylate the Cys289 residue of the Ang II type 1 receptor (AT1R), thus impairing AT1R:Ang II recognition [109,110]. Furthermore, in vivo studies show that SARS-CoV-2-infected rats treated with NO can restore the ACE2-angiotensin (1–7)-Mas system, outweighing the deleterious effects of COVID-19 progression by: (i) increasing circulating *S*-nitrosothiol levels: (ii) reducing vasoconstriction; and (iii) increasing aortic PKC nitrosylation [111,112]. COVID-19 mortality has been correlated to NO production in the endothelium and to NO bioavailability in different groups (e.g., age and gender) and/or in the presence of comorbidities. SARS-CoV-2 induces an inflammatory response mediated by various chemokines, cytokines, and other immune-related factors that in turn leads to endothelial dysfunction(s) such as a proliferative and prothrombotic status causing the disruption of the vascular integrity [113,114]. These conditions are associated with aging and cause the decrease in NO availability in the elderly [115,116,117]. Interestingly, these SARS-CoV-2-dependent inflammatory responses are attenuated in females, where estrogen generates a protective environment by increasing the expression and activity of the NOS-II enzyme and consequently of NO [118]. Therapeutic strategies based on NO donors and NOS inhibitors [31], as well as lifestyle factors (e.g., a diet rich in nitrate and physical exercise), have been proposed to restore the NO bioavailability in the host thus improving the health conditions of COVID-19 patients [32,98,99,100].

## 4. Effects of NO on Pathogenic Bacteria

**Figure 4:**
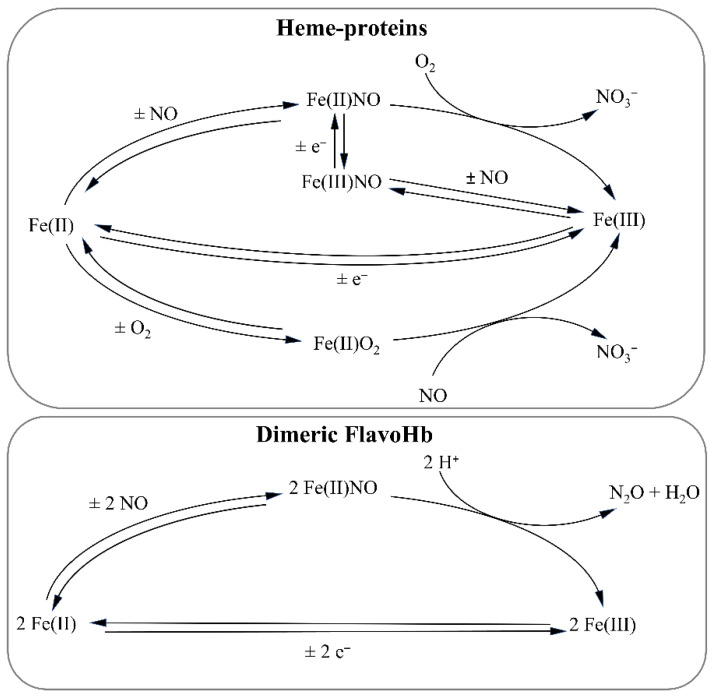
NO detoxification mediated by heme-proteins and dimeric Hmp. Heme-proteins include bacterial truncated hemoglobin, myoglobin, neuroglobin, and hemocyanin.

Physical and chemical barriers of the innate immune system generally protect the host from invading pathogens by activating macrophages that produce reactive oxygen species (ROS) (e.g., O_2_^●−^) and RNS, including NO. ROS and RNS can alter proteins, lipids, and nucleic acids of pathogenic microorganisms, leading to pathogen killing [119]. Interestingly, NO generated in the stomach participates in the protection against pathogens in addition to the stomach acidity [49,120]. However, some bacteria including *E. coli*, *S. typhimurium*, *Campylobacter jejuni*, *Mycobacterium leprae*, and *Mycobacterium tuberculosis* have evolved detoxification systems to protect themselves against host-induced oxidative and nitrosative stresses [48,49,121,122]. Interestingly, bacteria produce NO as a by-product of their own metabolism during anaerobic nitrate respiration [49] (Figure 4).

### 4.1. NO Detoxification in Enteric Bacteria

*E. coli* and *S. typhimurium* defend themselves against NO produced by the host immune system by expressing: (i) soluble Hmp flavohemoglobin (flavoHb); (ii) truncated hemoglobins (trHbs); (iii) di-iron-centered flavorubredoxin NorV and NADH-dependent oxidoreductase NorW (NorVW); and (iv) cytochrome *c* nitrite reductase NrfA [49,123,124,125,126]. FlavoHb expression and activity is NO-dependent [127,128,129,130]. Indeed, flavoHb displays an NO-dioxygenase activity, catalyzing the conversion of NO to NO_3_^−^ in the presence of O_2_ and NADH [131,132]. Moreover, flavoHb displays an NADH-dependent alkylhydroperoxide reductase activity, reducing several alkyl-hydroperoxides into their corresponding alcohols [130]. Furthermore, flavoHb has been suggested to repair lipid membrane oxidative damage generated during oxidative and nitrosative stress [126,130]. Under anoxic conditions, a flavoHb-dependent mechanism facilitating NO scavenging and reduction into N_2_O has been reported [127,128,129,130]. To date, flavoHbs have never been found in higher organisms [126,130].

While flavoHb can act both under oxic and anoxic conditions, leading to the production of NO_3_^−^ and N_2_O, respectively, NorVW and NrfA are active only under anaerobic or microoxic conditions, thus resulting in the most important enzymes acting in anaerobic NO detoxification [124,125,133]. While NorVW reduces NO to N_2_O, NrfA utilizes either NO or NO_2_^−^ to synthesize ammonia in O_2_-limited environments [49,124,125,133].

*E. coli* and *S. typhimurium* can survive in several different environments thanks to the expression of some transcriptional regulators, including NorR, NsrR, fumarate, nitrate reductase regulator (FNR), ferric-uptake regulator (Fur), and methionine repressor (MetR). The expression of these regulators ensures the response to NO [134,135,136,137,138,139]. FNR acts in the transition between aerobic and anaerobic growth and mediates the upregulation of several operons in response to nitrate and nitrite [136]. Fur controls the expression of genes implicated in the iron uptake, being especially crucial when iron availability is limited. Of note, Fur activity is also impaired by the presence of NO [49,139].

The enteric pathogen *C. jejuni* is exposed to a range of ROS and RNS produced by the host [140,141]. The ability of *C. jejuni* to detoxify RNS and ROS has been associated to the expression of a classic 3-on-3 globin (Cgb) and of a group III trHb (trHbP) [142,143,144,145,146]. Cgb helps the micro-aerophilic enteric microorganism to catalyze NO detoxification through a NO deoxygenase or denitrosylase mechanism that implies the transient formation of the heme-Fe(III)-ONOO^−^ complex [145,146]. Instead, TrHbP promotes primarily microaerobic growth and moderate respiration; moreover, it participates secondarily to NO metabolism [145,146]. Interestingly, ferrous trHbP (trHbP(II)) binds reversibly NO and displays nitrite reduction activity, leading to heme-Fe(II)-NO [147]. Moreover, ferric trHbP (trHbP(III)) binds reversibly to NO at low pH, whereas it undergoes reductive nitrosylation at alkaline pH. Furthermore, trHbP(III) cooperates with Cgb in the isomerization of peroxynitrite [148]. Of note, under high aeration conditions, *C. jejuni* strains defective for trHbP are disadvantaged compared to the wild-type ones, achieving lower growth yields and consuming O_2_ at approximately half the rate displayed by wild-type cells. Remarkably, trHbP mutants do not show increased sensitivity to NO or oxidative stress, suggesting that trHbP may play a role in cell respiration [142,143,144,147]. Overall, while Cgb plays a major role in the resistance to nitrosative stress and aerobically converts NO to nitrate [149], the contribution of trHbP is less prominent. Both Cgb and trHbP are devoid of NO-protective activity under O_2_-limited conditions that normally exist in vivo [150]. Therefore, the role of trHbP is distinct from that of the Cgb that is involved in O_2_ metabolism [142,144], likely performing a peroxidase- or P450-like oxygen chemistry [151].

### 4.2. NO Detoxification in M. leprae and M. tuberculosis

*M. leprae* and *M. tuberculosis* represent two of the most dangerous infective pathogens for humans [48,152,153,154]. The ability of mycobacteria to persist in vivo in the presence of RNS produced by activated macrophages [155,156] implies the existence of pseudo-enzymatic detoxification systems, including trHbs [47,48,121,122,157,158,159,160,161,162,163].

The intracellular pathogen *M. tuberculosis* expresses genes encoding for trHbN (belonging to group I) and trHbO (belonging to group II). TrHbN has primarily been linked to NO detoxification, while trHbO has been proposed to be involved in O_2_ uptake/transport and/or redox sensing [122,157,164,165,166,167]. *M. leprae* expresses only trHbO (i.e., GlbO), which shows both O_2_ uptake/transport and NO detoxification properties [47,121,161].

The involvement of *M. tuberculosis* trHbN in the protection against RNS has been demonstrated in vivo using both reverse genetic approaches and homologous or heterologous expression systems [158,159,163]. Indeed, the *Mycobacterium bovis* mutant lacking trHbN does not oxidize NO to NO_3_^‒^ and shows decreased respiration upon exposure to NO [158]. A similar behavior has been predicted for *M. tuberculosis* given the close phylogenetic relationship between *M. bovis* and *M. tuberculosis* and the high identity of trHbNs expressed in these two neighbor species [162]. Moreover, the heterologous expression of *M. tuberculosis* trHbN significantly protects both *M. smegmatis* and a flavoHb-deprived mutant of *E. coli* from NO damage through an O_2_-sustained detoxification mechanism [159]. A similar protective effect has also been reported for *M. smegmatis* trHbN [163]. Lastly, the overexpression of *M. leprae* trHbO alleviates the growth inhibition caused by NO donors in the *E. coli* hmp mutant, partially complementing the defect in flavoHb synthesis [48,121]. Both *M. tuberculosis* trHbN and *M. leprae* GlbO catalyze peroxynitrite scavenging, allowing mycobacteria to survive also in the adverse host macrophagic environment [47,48]. The structural and functional characterization of nitrobindin (Nb) from *M. tuberculosis* highlighted its ability to scavenge peroxynitrite. This supports the notion that Nb can be part of the pool of proteins required to scavenge RNS produced by the host during the immune response. In this framework, *M. tuberculosis* Nb may become a novel therapeutic target for the treatment of *M. tuberculosis* infections as reported also for mycobacterial trHbN [47,48,168,169,170,171,172,173,174,175,176].

### 4.3. Lysteria Monocytogenes Escapes from NO

The Gram-positive *Lysteria monocytogenes* is an intracellular pathogen implicated in several outbreaks of foodborne diseases (e.g., gastroenteritis, meningitis, and abortion in susceptible individuals) [51,177]. Upon ingestion, *L. monocytogenes* survives in TLR-activated macrophages by escaping its internalization via the action of the pore-forming toxin listeriolysin O (LLO) [178]. The maturation of the phagosome requires the activation of the vacuolar H^+^-ATPase (V-ATPase) that leads to phagosomal acidification through the loss of the early endosomal marker Rab5 and by acquisition of the lysosomal membrane protein-1 (LAMP-1) [50,51]. Once in the cytosol, *L. monocytogenes* induces actin polymerization via the surface protein ActA to form pseudopod projections that propel bacteria from a primary infected donor cell to a secondary uninfected recipient cell via a process known as cell–cell spread [51]. This process allows the pathogens to remain intracellular, thus avoiding extracellular defense mechanisms and humoral immune factors [177].

*L. monocytogenes* takes advantage of the NO produced in macrophages by NOS-II in response to TLR to enhance the cell–cell spread during systemic infection [50,51]. In detail, NO delays the maturation of secondary vacuole-containing membrane-encapsulated bacteria, thus increasing both the percentage of infected recipients and the number of bacteria per recipient cell. Although the NO-based mechanism that selectively attenuates secondary vacuole maturation is still unclear, NO produced by NOS-II inhibits the V-ATPase, which could delay maturation of secondary vacuoles [179,180]. However, the inhibition of V-ATPase by bafilomycin and concanamycin A does not reverse NO effects. For this reason, it has been speculated that NO reduces the phagosome acidification and degrades secondary vacuoles by direct modification of Rab4 and LAMP-1 proteins or indirect activation of protein kinase G [51,181]. Of note, during cell–cell spread the effects of TLR stimulation with LPS prevail on the antibacterial action of IFN-γ. Indeed, even if the amount of NO produced in LPS- and IFN-γ-stimulated macrophages is comparable, the TLR stimulation of “recipient” macrophages with IFN-γ alone is insufficient to reduce the *L. monocytogenes* spreading. TLR signals induced by IFN-αβ, IL-6, or TNF-α inhibit the induction of IFN-γ antimicrobial effectors (e.g., p65 GTPases) impairing *L. monocytogenes* killing in both primary and secondary vacuoles [50,51]. Furthermore, *L. monocytogenes* spread is organ specific. Indeed, NOS-II inhibitors partially prevent bacterial spread in the liver but not in the spleen, possibly because NOS-II is significantly less expressed in the spleen compared to the liver [50,51].

## 5. Antiparasitic Effects of NO on Protozoa and Metazoa

NO exerts antiparasitic effects on Protozoa (i.e., *Leishmania*, *Trypanosoma*, *Giardia*, *Trichomonas*, *Naegleria*, *Entamoeba*, *Plasmodium*, *Toxoplasma*, and *Babesia*) and Metazoa (i.e., *Schistosoma*, *Fasciola*, *Dicrocoelium*, *Opisthorchis*, *Taenia*, *Echinococcus*, *Trichinella, Ascaris*, and *Onchocerca*). Interestingly, a role of host heme-proteins (i.e., myoglobin (Mb), neuroglobin (Ngb), Hb, and hemocyanin (Hc)) has been postulated in *Trypanosoma, Toxoplasma, Plasmodium*, and *Schistosoma* protection from the parasiticidal effect of NO [26,46,182,183,184] (Figure 4).

### 5.1. Trypanosoma cruzi

*T. cruzi* is the protozoan parasite that causes the Chagas disease. *T. cruzi* shows a complex life cycle, involving the triatomine hematophagous vector and a mammalian host. In the host, trypomastigotes penetrate phagocytic cell lines, transform into the amastigote stage, which replicate, and emerge from ruptured cells as trypomastigotes. Then, while some of the parasites penetrate again in cells continuing with the intracellular division, other trypomastigotes circulate in the blood to be picked up by triatomine bugs during the blood meal [185,186].

NO blocks the *T. cruzi* life cycle both in vitro and in vivo [187,188]. Macrophages from mice infected by *T. cruzi* kill trypomastigotes by producing high levels of NO [189,190,191]. Furthermore, cardiomyocytes invaded by *T. cruzi* express NOS isoforms, thus increasing NO levels and metabolites [192,193,194]. Accordingly, mice deficient in NOS-II [195] or treated with NOS-II inhibitors show an increased susceptibility to *T. cruzi*. In addition, mice infected with *T. cruzi* and exposed to NO donors are more protected against trypomastigotes compared to the control group [196,197]. Lastly, NO promotes splenocyte apoptosis during the acute phase of *T. cruzi* infection in mice [198,199] and modulates parasite cell entry, thus contributing to the pathogenesis of Chagas cardiomyopathy [188,200,201,202].

In mammals, *T. cruzi* preferentially invades heart as well as skeletal and smooth muscle [185,186] where Mb acts as a NO scavenger [182]. In fact, oxygenated Mb (Mb(II)O_2_) reacts rapidly and irreversibly with NO leading to the harmless NO_3_^−^ and the ferric Mb derivative (Mb(III)) [203]. In turn, Mb(III) is reduced to the physiologically active form by Mb reductase [204,205]. In heart and skeletal muscle cells, the high concentration of Mb (~3 × 10^−4^ M) could capture NO thus reducing NO-related adverse effects (e.g., inhibition of the respiratory chain of the parasite) towards *T. cruzi* [182]. Of note, it has been also speculated that *Trypanosoma brucei* preferentially localizes in the brain areas expressing Ngb, a hemeprotein involved in the NO/O_2_ metabolism [182,206].

### 5.2. Toxoplasma gondii

The preferential localization in retina, heart, and skeletal muscle cells of *T. gondii* may reflect the NO scavenging activity of Ngb and Mb [207], whose concentrations are ~1.0 × 10^−3^ M [203] and ~3.0 × 10^−4^ M, respectively [204]. Ngb and Mb inactivate efficiently NO, reducing its toxoplasmacidal effect and favoring *T. gondii* colonization in retina and muscle [207,208,209,210]. In contrast to mycobacterial pathogens, *T. gondii* does not express trHbs as a protection mechanism against NO [207,211]. However, *T. gondii* encodes superoxide dismutase, catalase, glutathione/thioredoxin-like peroxidases, and peroxiredoxins that all participate to NO metabolism [212].

### 5.3. Plasmodium falciparum

Oxygenated Hb (Hb(II)O_2_) has been postulated to protect intraerythrocytic *Plasmodia* from the parasiticidal effect of NO [213]. *P. falciparum* is the etiological agent of malaria, the most important cause of death due to a vector-borne infectious disease. Its life cycle involves two hosts (i.e., humans and the *Anopheles stephensi* mosquito) and several developmental stages in each one. Human infection with *P. falciparum* starts when, during the blood meal, a female *Anopheles* mosquito injects sporozoites in the host peripheral circulation. The parasite reproduces asexually in the liver cells (exoerythrocytic schizogony) and in red blood cells (RBCs) (erythrocytic schizogony) and then develops into sexual precursors (gametocytes), which can be taken up by mosquitoes in a blood meal to complete the life cycle [214]. In RBCs, the parasite is surrounded by Hb which acts as a potent scavenger of NO by blocking its antiparasitic effects [215,216]. In vivo, Hb may operate a dual role, both as a scavenger and as an NO donor. Thus, when RBCs are saturated with oxygen, Hb(II)O_2_ reacts with NO, preventing the antiparasitic effect. In contrast, at low oxygen tension, Hb(II) readily releases NO that exerts cytotoxic effects towards *P. falciparum* [213,217,218].

### 5.4. Schistosoma

Schistosomiasis is a devastating parasitic disease diffused in tropical and subtropical countries [219]. Eggs deposited in water release a free-swimming ciliated larva (miracidium) able to penetrate a freshwater snail host. In the snail tissues, miracidia transform into mother sporocysts that generate daughter sporocysts. The latter, after migration into the digestive gland, give rise to infectious fork-tailed larvae called cercariae. These larvae are shed from the snail and penetrate the human skin. During skin penetration, larvae lose tails and become schistosomulae that migrate via the venous circulation to the lungs and the heart. Then, they develop in the liver, exiting the liver via the portal vein system when mature. Male and female adult worms migrate to mesenteric venules of the bowel/rectum and venous plexus of the bladder, depending on the species. Females deposit eggs that move progressively toward the lumen of intestine and of both bladder and ureters. Lastly, eggs are eliminated with feces or urine, the life cycle starting again [219,220].

RNS affect the *Schistosoma* life cycle at different stages [28,46,183,219,220,221]. Hemocytes circulating in the hemolymph of resistant strains of the freshwater snail host *Biomphalaria glabrata* eliminate sporocysts of *Schistosoma mansoni* through inducible NO. Accordingly, NO synthase inhibitors (e.g., N^ω^-nitro-L-arginine methylester) and the NO scavenger 2-(4-carboxyphenyl)-4,4,5,5-tetramethylimidazoline-1-oxyl-3-oxide) reduce hemocyte-mediated killing of *S. mansoni* sporocysts [222]. Although NO-mediated cytotoxicity of macrophages is known to eliminate the schistosomula of *S. mansoni* [221,223,224], the skin-stage schistosomula can penetrate the host skin [225]. Thus, skin-stage schistosomula of *Schistosoma japonicum* can evade the macrophage NO-mediated cytotoxicity by Sj-Ca8, a calcium-binding protein expressed in cercariae, skin-stage schistosomula, lung-stage schistosomula, and adult worms [225]. Sj-Ca8 impairs skin penetration by cercariae, suppresses macrophage migration, and impairs NO release [225]. These findings render Sj-Ca8 a potential vaccine candidate and a chemotherapeutic target for the prevention and treatment of schistosomiasis [225].

The survival of schistosomula and of adult worms in the definitive host (e.g., human) and of sporocysts in the intermediate freshwater snail host has been proposed depend on the role of Hb and Hc, respectively. Both proteins impair the antiparasitic effects of NO [183]. In the case of Hc, the binuclear oxygenated metal center of Hc (Cu(II)-O_2_^2−^-Cu(II)) reacting with NO, generates harmless NO oxidation products (i.e., NO_2_^−^) and the Cu(I)-Cu(II) complex [183]. In the mammalian host, although anemia occurs during schistosomiasis, the high concentration of Hb(II)O_2_ intercepts NO and protects the parasite [183].

### 5.5. Ascaris lumbricoides and Ascaris suum

*A. lumbricoides* and *A. suum* are nematodes able to parasitize the intestine of humans and pigs, respectively [226,227,228,229]. Ascariasis occurs when embryonated eggs that contaminate hands, utensils, or food are swallowed. In the small intestine the eggs hatch, releasing the larvae that go through the intestinal wall and migrate through the liver and heart, up to lungs. In the lung, larvae are expectorated and ingested, thus arriving to the small intestine, where they mature into adult worms and produce new eggs which are expelled with feces, contaminating the environment [229].

**Figure 5:**
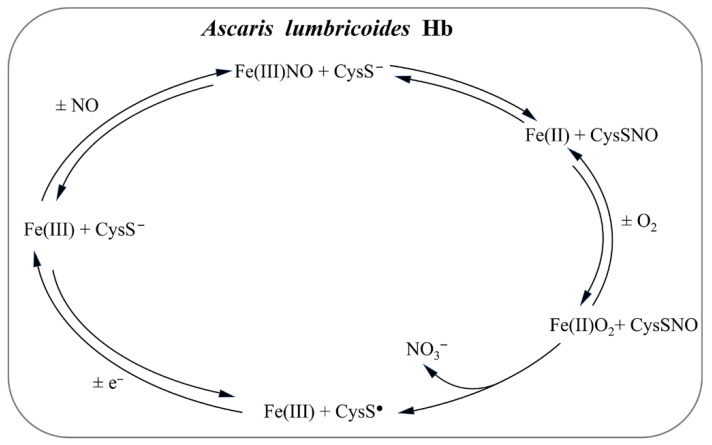
NO detoxification mediated by *A. lumbricoides* Hb.

*Ascaris* worms express an octameric Hb that acts as an NO-dependent deoxygenase by using the endogenous NO as a substrate to detoxify O_2_ (Figure 5) [227,228]. Although the primary function of *Ascaris* Hb is to remove O_2_ from the perienteric fluids, it may also protect the nematode against the NO present either in the host gut or generated by the host innate immune response [227,228]. The CysE15 residue, located in the distal side of the *A. lumbricoides* Hb heme-pocket, plays a key role in NO destiny, allowing the NO-mediated enzymatic consumption of O_2_ [228]. On the other hand, the CysF9 residue, located in the proximal side of the heme-pocket of most tetrameric mammalian Hbs, allows the NO-mediated control of O_2_ delivery [230,231,232,233]. Therefore, *A. lumbricoides* Hb has been postulated to be evolutionary positioned between the primordial bacterial flavoHbs catalyzing NO/SNO detoxification and cooperative mammalian tetrameric Hbs, which display an NO-mediated O_2_ delivery mechanism [227,228] (Figure 5).

## 6. Effects of NO on Fungal Infection

The mushroom kingdom comprises about 1.5 million species, but only 400 fungal species are pathogenic to humans [234]. Fungal pathogens, including *Candida albicans* and *Cryptococcus neoformans*, developed several mechanisms to elude the host immune defenses [234,235,236]. Since many stress-protective enzymes use iron as a cofactor, pathogenic fungi induce the expression of genes involved in iron acquisition in response to RNS [234].

*C. albicans* is part of the healthy human microbiota and colonizes several niches in the body (e.g., oral cavity, gastrointestinal tract, female reproductive tract, and skin) [237]. In healthy individuals, *C. albicans* represents a harmless commensal that coexists in harmony with other members of the microbiota. However, alterations in the host immune system, pH variations in the local environment, and/or antibiotic therapy can favor *C. albicans* proliferation and infection [234]. *C. albicans* can tolerate high levels of NO produced by the host immune response through the expression of the inducible flavoHb-related protein YHB1, which plays a critical role in NO metabolism and in RNS detoxification [235]. YHB1 acts as an NO dioxygenase by converting NO to harmless nitrate [234]. The induction of YHB1 expression is mediated by Cta4p, a Zn(II)2-Cys6-DNA-binding protein belonging to a family of fungal transcription factors [238].

The virulence of *C. neoformans* is due to the FHB1 flavoHb, which is involved in the detoxification of NO produced by the host-inducible NOS-II [238].Under nitrosative stress, *C. neoformans* activates the synthesis of proteins involved in detoxification mechanisms including chaperones, oxidoreductase, thioredoxin reductase, and dehydrogenase [232,234].

## 7. Conclusions

RNS exert positive antiviral effects. This is supported by the success of NO-based therapies as well as by lifestyle factors that restore the physiological NO levels and consequently improve the clinical settings of patients affected by respiratory infections. Indeed, boosting NO, which is depleted by psychological stress and viral assault, provides protection against viral proliferation. Notably, the FDA approved inhaled NO for the treatment of pulmonary hypertension, thrombocytopenia, and respiratory infections, including COVID-19.

**Figure 6:**
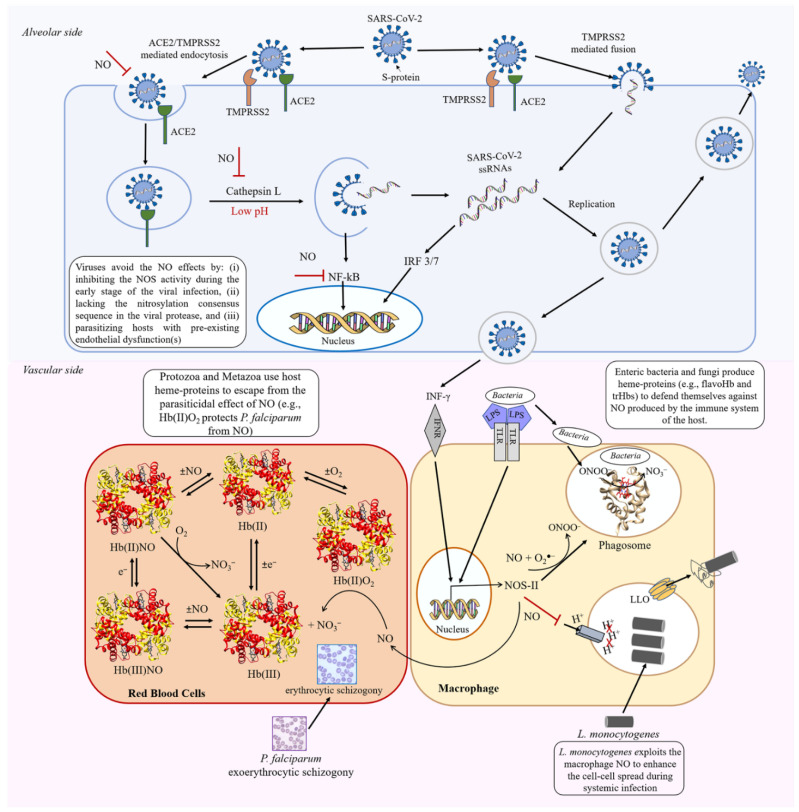
Overview of pathogen strategies to escape from NO-based host defense.

In bacteria, protozoa, metazoa, and fungi, the expression of hemeproteins is at the root of several reactions involving NO. This suggests that the evolution of hemeproteins has been greatly influenced by NO-related functions and, in some cases, by mechanisms required to escape from NO-related toxic effects. Indeed, NO is part of denitrifying bacteria metabolism as an intermediate of the nitrogen cycle. However, NO is toxic and harmful for those microorganisms that have not developed mechanisms to counteract host-produced NO effects. Several trHbs found in bacteria are implicated in the tolerance to nitrosative stress; moreover flavoHb has been shown to protect pathogens against NO both aerobically and anaerobically (Figure 6). Since NO can exert beneficial or detrimental functions, further studies are required to understand the evolutionary adaptations of pathogens to counteract host-produced nitrogen and oxygen reactive species.

## Data Availability

Not applicable.
